# Is Nursing a Profession?

**Published:** 1919-02-15

**Authors:** 


### IS NURSING A PROFESSION?

Apart from all minor or petty considerations, the question whether or not nursing may be included amongst the professions is worth discussing. Accuracy in terminology is of far-reaching importance. Words are often used with the object of concealing or obscuring the truth, and people's attitude and actions are not infrequently determined by their outlook. There is something to be said in favour of calling a spade a spade. The word "profession" is used in a variety of senses. Any occupation followed for a livelihood may be correctly described as a profession. It is used also to distinguish the amateur from the artist, as in the case of a thief. But there are also employments which convention classes as professions, and amongst these it is usual to include nursing. I take the liberty of raising the question whether such usage is or is not justified, and I do so because I regard the answer as being of more than passing importance.

### What is a Profession?

Perhaps the best way to begin is to ascertain and set out clearly what are the distinctive characteristics of a profession. What are the symptoms? It is like a doctor diagnosing a disease, or a botanist classifying a plant. If a man buys commodities and sells them at a profit he is said to be engaged in commerce. If a worker in stone, or wood, or iron, he is a craftsman. Similarly, I think, it will be found that an occupation or employment correctly described as a profession requires that a person who follows it should spend a period of years without remuneration in pupilage. Five years or thereabouts is the period required for medicine or the law. Dentistry, accountancy, and architecture apprenticeships are somewhat shorter. The British Admiralty consider it necessary to begin the training of an officer before the boy enters his teens. There are variations, but I think it will be found in every instance that the entrance to professional life is an unpaid period of study and training. A man or woman may acquire the necessary skill for any profession in their own way and according to their own ideas. A self-taught physician may conceivably know more of the art of healing than a man from the University. But if his name be not graven on the white stone he is, in the eyes of the law, a quack. He does not belong to the profession!

### A "Bribes" Policy for Probationers.

I think it may be found somewhat difficult to arrive at a satisfactory conclusion as to whether or not the trained nurse can, in the sense indicated, claim to belong to a recognised profession. The subject is of more than passing importance and demands clear and profound thinking. For of late years the whole trend of hospital committees has been to degrade the status of the nurse. This has been done unconsciously but consistently, and the root of the evil is economic. When there is a shortage of probationers the policy adopted is to offer better wages. This clumsy hand-to-mouth remedy tends, in the long run, to the degradation of the nurse by removing the probationer from what I may call a condition of pupilage, and making of her a wage-earner, not to say a drudge. The whole subject of probation requires careful investigation, and I venture to suggest that it ought to form an important part of the duty of the Committee of Enquiry which, I understand, the College of Nursing proposes to appoint. Probably many will not agree with me that probationers ought to be permitted to enter on their course of training at the age of eighteen. This is the school-leaving age—the age for beginning a career or for entering a university. The reason why there is a consensus of contrary opinion is because those who have considered the subject have never regarded the probationer merely as a pupil. She is, in actual fact, as well as being a pupil, a hospital hand—a wage-earner—a useful person—a cheap servant. And, therefore, whenever the supply of probationers diminishes hospital committees respond by offering a few pounds more per annum as an inducement. This is not done in the professions, and the effect on the nursing world has been to lower rather than to raise the standard—to co-ordinate nursing with domestic service rather than to bring it into line with the higher and more intellectual employments.

### An Invigorating Place in the Sun.

If ever nursing is to be given a high status and given an invigorating place in the sun, the transformation must begin with the probationer. She must cease to be a drudge and become more and more a student. It is in the after years that the nurse wants pay and prospect, and if this is assured there need be no raising of probationers' pay, nor, indeed, any pay at all for them. I am constantly told by those who know that it is impossible for probation to begin at eighteen. Under existing conditions this is obvious. No young and growing girl unprovided by nature with a cast-iron frame could possibly face the existing conditions of probation. The prescribed period of training would make of her a physical wreck. If, however, she is no longer regarded as a hospital hand, but rather as an apprentice to a profession, I fail to see why the minimum age-limit should not be substantially reduced, nor why the trained nurse should not be able to begin her professional career at the same age as a doctor. Do you?

